# Supplementary material for: Causal Relationship between Aging and Anorexia Nervosa: A White-Matter-Microstructure-Mediated Mendelian Randomization Analysis
Source: Biomedicines. 2024 Aug 16;12(8):1874. doi: 10.3390/biomedicines12081874 (PMC11351342; doi:10.3390/biomedicines12081874)
Supplement: Supplementary file 1 [file biomedicines-12-01874-s001.zip › Supplementary Figure/Figure S4/Singe SNP plot of MR analysis for TL to ubm-b-1883.pdf]

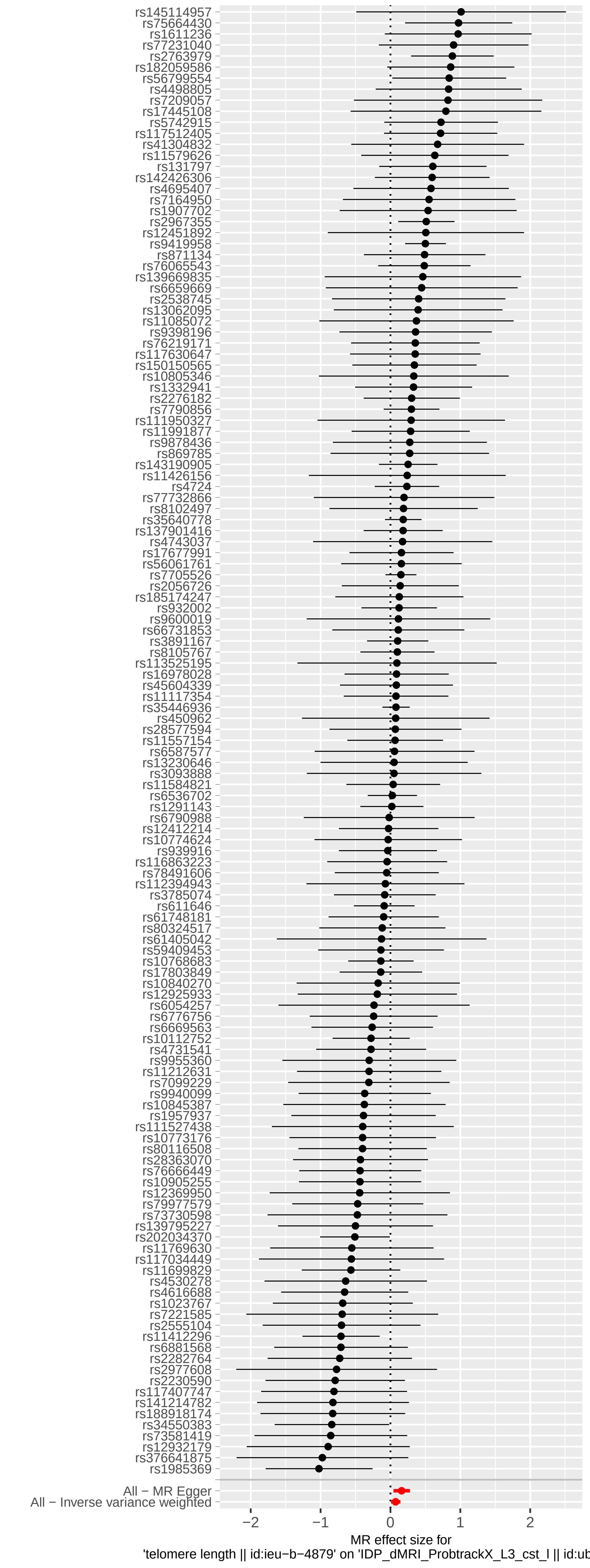
rs145114957
rs75664430
rs1611236
rs77231040
rs2763979
rs182059586
rs56799554
rs4498805
rs7209057
rs17445108
rs5742915
rs117512405
rs41304832
rs11579626
rs131797
rs142426306
rs4695407
rs7164950
rs1907702
rs2967355
rs12451892
rs9419958
rs871134
rs76065543
rs139669835
rs6659669
rs2538745
rs13062095
rs11085072
rs9398196
rs76219171
rs117630647
rs150150565
rs10805346
rs1332941
rs2276182
rs7790856
rs111950327
rs11991877
rs9878436
rs869785
rs143190905
rs11426156
rs4724
rs77732866
rs8102497
rs35640778
rs137901416
rs4743037
rs17677991
rs56061761
rs7705526
rs2056726
rs185174247
rs932002
rs9600019
rs66731853
rs3891167
rs8105767
rs113525195
rs16978028
rs45604339
rs11117354
rs35446936
rs450962
rs28577594
rs11557154
rs6587577
rs13230646
rs3093888
rs11584821
rs6536702
rs1291143
rs6790988
rs12412214
rs10774624
rs939916
rs116863223
rs78491606
rs112394943
rs3785074
rs611646
rs61748181
rs80324517
rs61405042
rs59409453
rs10768683
rs17803849
rs10840270
rs12925933
rs6054257
rs6776756
rs6669563
rs10112752
rs4731541
rs9955360
rs11212631
rs7099229
rs9940099
rs10845387
rs1957937
rs111527438
rs10773176
rs80116508
rs28363070
rs76666449
rs10905255
rs12369950
rs79977579
rs73730598
rs139795227
rs202034370
rs11769630
rs117034449
rs11699829
rs4530278
rs4616688
rs1023767
rs7221585
rs2555104
rs11412296
rs6881568
rs2282764
rs2977608
rs2230590
rs117407747
rs141214782
rs188918174
rs34550383
rs73581419
rs12932179
rs376641875
rs1985369
All − MR Egger
All − Inverse variance weighted
−2
−1
0
1
2
MR effect size for
'telomere length || id:ieu−b−4879' on 'IDP_dMRI_ProbtrackX_L3_cst_l || id:ub
